# Solitary pulmonary metastasis after meningioma surgery of the head: a case report

**DOI:** 10.1186/s40792-022-01379-9

**Published:** 2022-02-05

**Authors:** Takahiro Utsumi, Tomohito Saito, Mitsuaki Ishida, Natsumi Maru, Hiroshi Matsui, Yohei Taniguchi, Haruaki Hino, Tomohiro Murakawa

**Affiliations:** 1 Department of Thoracic Surgery, Kansai Medical University Hospital, 2-3-1 Shin-machi, Hirakata, Osaka, 573-1191 Japan; 2 Department of Pathology and Laboratory Medicine, Kansai Medical University Hospital, Osaka, Japan

**Keywords:** Atypical meningioma, Metastatic meningioma, Pulmonary metastasis, Surgical resection

## Abstract

**Background:**

Meningioma is the most common type of benign primary brain tumor that is rarely associated with distant metastasis. No established treatment strategy for metastatic meningiomas exists to date. Herein, we report a case of solitary pulmonary metastasis of meningioma detected 2 years after neurosurgical resection of the primary tumor.

**Case presentation:**

A 75-year-old male patient underwent neurosurgical resection of a convexity meningioma (World Health Organization grade II atypical meningioma), followed by postoperative radiotherapy for the residual tumor. Two postoperative years later, a solitary 10-mm pulmonary nodule in the left lower lung lobe was detected on chest computed tomography. The patient underwent video-assisted thoracoscopic left lower lobectomy for suspected pulmonary metastasis of meningioma. The pathological diagnosis was solitary pulmonary metastasis of meningioma. No sign of further recurrence was noted at 8 months postoperatively.

**Conclusions:**

We present a rare and unique surgical case of solitary pulmonary metastasis of meningioma. Further investigation is necessary to establish the standardized treatment strategy for metastatic meningiomas.

## Background

Meningioma is the most common type of primary brain tumor, accounting for approximately 30% of all primary brain tumors [1]. Although meningiomas are usually associated with a benign clinical course, they can sometimes show malignant behavior, such as distant metastasis. Although only a few cases of pulmonary metastasis of meningioma have been reported to date, the lungs have been described as the most frequent site of meningioma metastasis [2]. We herein report a unique and rare surgical case of metastatic meningioma presenting with solitary pulmonary metastasis 2 years after neurosurgical resection of the primary tumor.

## Case presentation

**Fig. 1:**
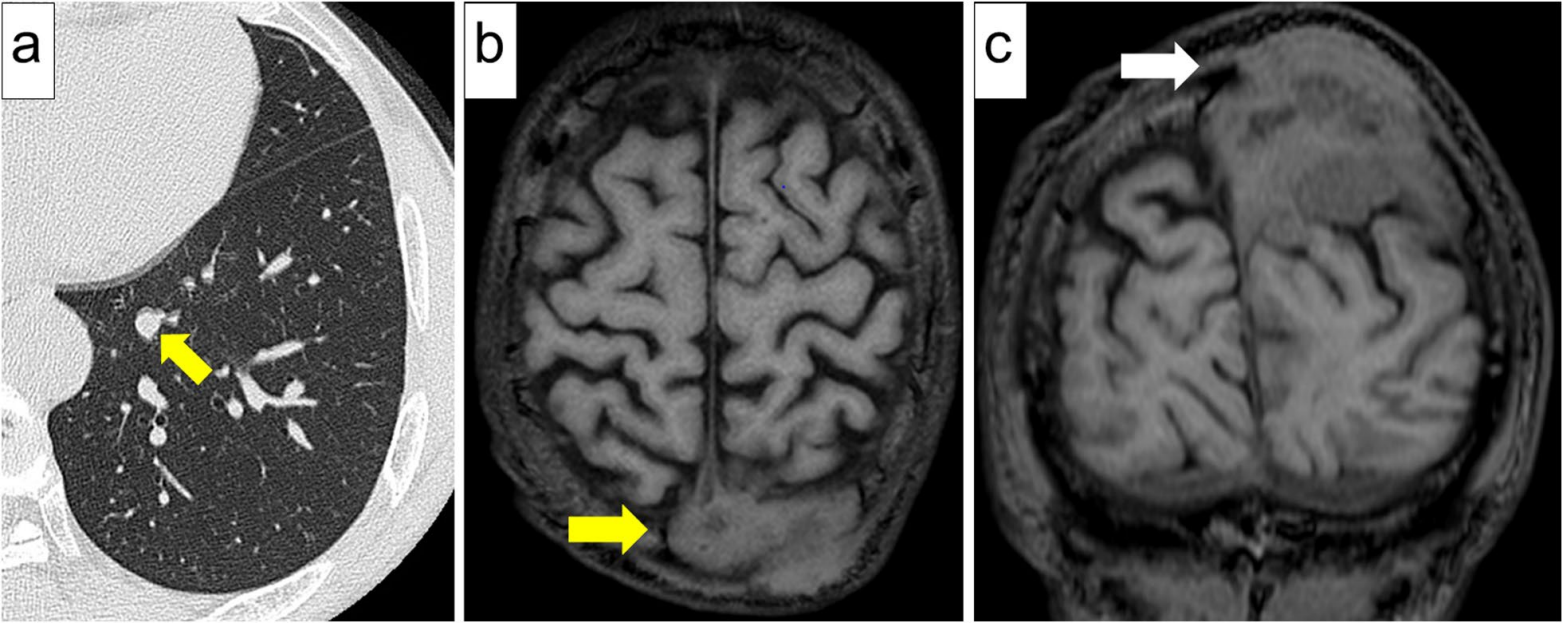
Medical imaging findings. **a** Computed tomography of the chest showing a 10-mm solitary pulmonary nodule in the left lower lobe of the lung (yellow arrow). **b** Two years before the referral, magnetic resonance imaging revealing a 65-mm tumor in the left parietal region (yellow arrow, axial section) and **c** skull infiltration of the tumor (white arrow, coronal section)

**Fig. 2:**
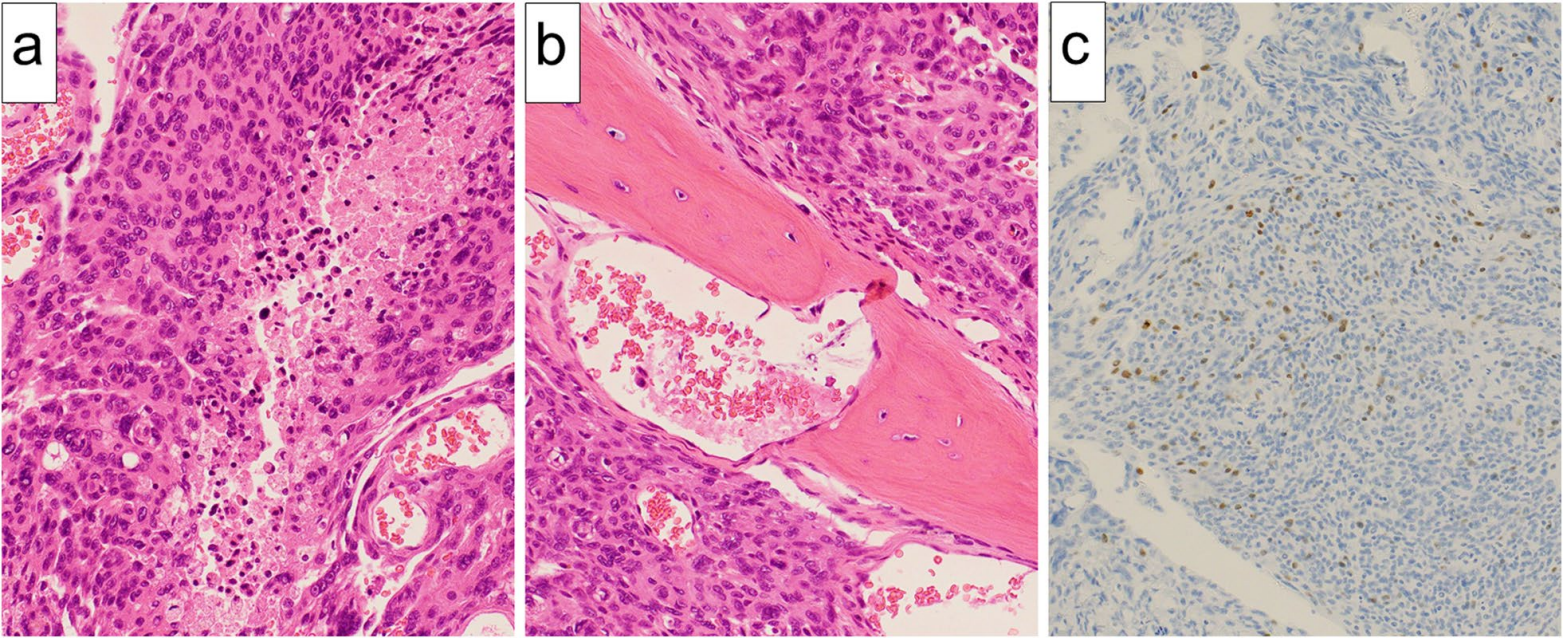
Pathological findings of the primary intracranial meningioma. **a** Short spindle-shaped tumor cells with acidophilic cytoplasm were densely proliferated, and the area of necrotic tumor cells were also confirmed (hematoxylin–eosin staining, original magnification 200×). **b** Tumor cells were observed to infiltrate the bone tissue (hematoxylin–eosin staining, original magnification 200×). **c** Ki-67 staining with MIB-1 antibody stained 10% of tumor cells (immunostaining with anti-MIB-1 protein polyclonal antibody, original magnification 100×)

A 75-year-old male patient with a 0.5 pack-year smoking history was referred our department for further investigation of a solitary nodule in the left lower lung lobe (Fig. 1a). The patient underwent craniotomy for neurosurgical resection of the left convexity meningioma 2 years before the referral (Fig. 1b, c). The surgery resulted in incomplete resection with a residual tumor around the superior sagittal sinus. Thus, the patient subsequently underwent postoperative radiotherapy for the residual tumor. The pathological diagnosis was World Health Organization (WHO) grade II atypical meningioma [3]. The lesion showed an MIB-1 labeling index of 10% (Fig. 2a–c). Of note, chest computed tomography scan was not performed prior to or at the time of neurosurgical resection of the primary meningioma.

**Fig. 3:**
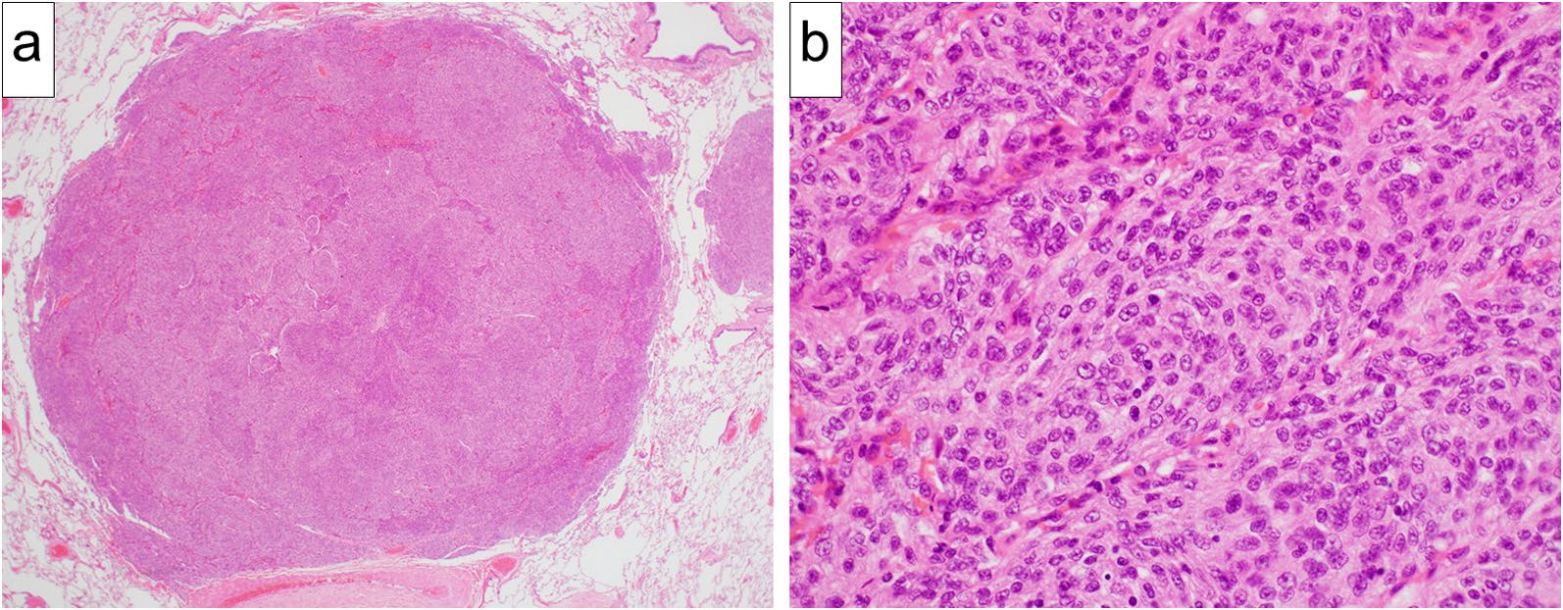
Pathological findings of the pulmonary metastasis of meningioma. **a** The boundary between the tumor and surrounding tissue was clear (hematoxylin–eosin staining, original magnification 20×). **b** Short spindle-shaped tumor cells with a slightly acidophilic cytoplasm and exhibiting complex proliferation were detected (hematoxylin–eosin staining, original magnification 400×)

Chest computed tomography at our department revealed a solitary 10-mm nodule in the left lower lobe of the lung. Neither intracranial recurrence nor distant metastasis other than the left lung was detected. The patient’s clinical history led us to suspect solitary pulmonary metastasis of meningioma, considering stage I primary lung cancer as a potential differential diagnosis. The patient finally underwent video-assisted thoracoscopic left lower lobectomy. Pathological investigation revealed short spindle-shaped tumor cells with clear margins that exhibited complex proliferation (Fig. 3a, b). According to the WHO classification of tumors, pulmonary metastasis of meningioma is histologically identical to primary pulmonary meningioma, which by definition does not involve lesions in the central nervous system [4]. As the patient in the current case had a neurosurgical history of left convexity meningioma, the risk of primary pulmonary meningioma was ruled out. Thus, the final pathological diagnosis was solitary pulmonary metastasis of meningioma. The patient was discharged from the hospital on the 11th postoperative day without any complication. No sign of further recurrence was noted at 8 months postoperatively.

## Discussion

Meningioma accounts for approximately 30% of all primary brain tumors [1], frequently affecting female patients aged between 20 and 60 years. The supratentorial region is the most common site of meningioma involvement, followed by the infratentorial region and the spinal cord [5]. According to the WHO histological classification of meningiomas, grade I indicates low-grade meningioma; grade II, intermediate grade meningioma; and grade III, high-grade meningioma [3]. Most cases (90.0%–94.3%) are classified as grade I, 4.7%–7.2% as grade II, and 1.0%–2.8% as grade III [6].

In addition to the WHO histological grade, the MIB-1 labeling index [7] has recently been reported to be associated with local recurrence and prognosis. In general, the cell-division potential is considered high when the MIB-1 labeling index is ≥ 5%, and the risk of meningioma-related mortality increases when the MIB-1 labeling index of the primary lesion was ≥ 19.2% [8].

The first choice of treatment for primary intracranial meningioma is neurosurgical resection. Radiotherapy is usually considered for patients with incompletely resected tumor, those with local recurrence, and inoperable patients [5]. Distant metastasis is rare and found in only 0.15%–0.76% of patients with primary intracranial meningioma [8]. The lung is the most common site of meningioma metastasis, followed by the bones and liver [2]. Pulmonary metastasis of meningioma accounts for 60% of the cases of metastatic meningiomas [9, 10], presenting more frequently as multiple pulmonary metastases than solitary pulmonary metastasis [2].

Metastasis of meningioma could be explained by hematogenous metastasis in most cases, but some could be explained by seeding in the central nervous system via the cerebrospinal fluid [11, 12]. Histologically, pulmonary metastasis of cranial meningioma is identical to primary pulmonary meningioma, a rare extracranial meningioma [4], with only 53 cases reported to date [13, 14]. Thus, the detection of meningioma in the central nervous system is a key for obtaining differential diagnosis. Risk factors for distant metastasis of meningioma include history of open cranial neurosurgery (i.e., inflow of tumor cells into the bloodstream or cerebrospinal fluid during the surgical procedure), venous sinus infiltration, local recurrence, and histological malignancy [8]. Our patient had multiple risk factors for distant metastasis of meningioma: a history of open cranial neurosurgery, superior sagittal sinus infiltration, and histology of WHO grade II atypical meningioma.

**Fig. 4:**
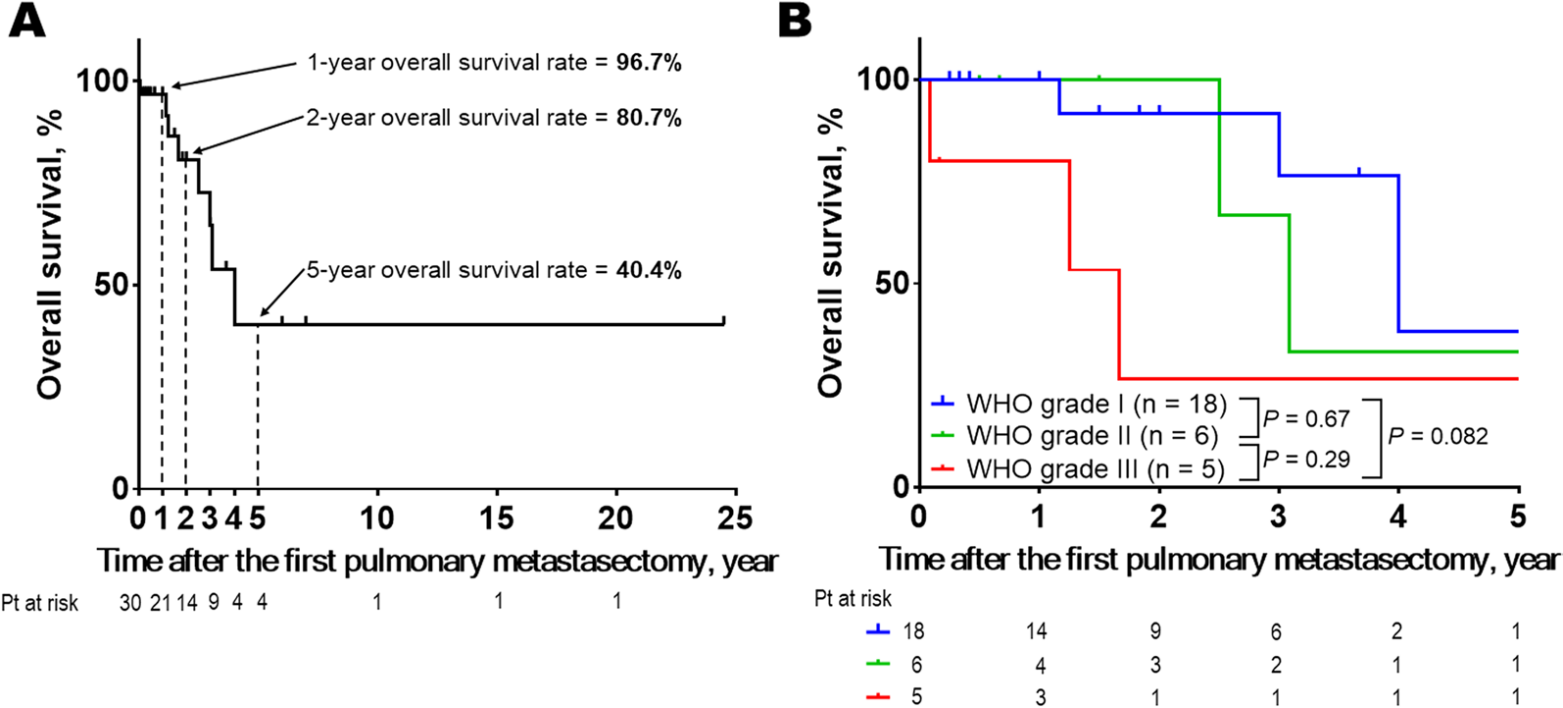
Overall survival after the first pulmonary metastasectomy for lung metastasis of cranial meningioma based on the integration of the reported cases (*n* = 30) [5, 6, 15, 17–38]. **a** The 1-, 2-, and 5-year overall survival rates based on the integration of all reported cases (*n* = 30) were 96.7%, 80.7%, and 40.4%, respectively. **b** WHO grade III meningioma was more likely to be associated with worse overall survival than WHO grade I meningioma (*P* = 0.082). The overall survival did not significantly differ between patients with WHO grade I and II meningioma (*P* = 0.67) and between patients with WHO grade II and III meningioma (*P* = 0.29, 0.082)

Consequently, no standardized treatment strategy for pulmonary metastasis of meningioma has been established. Specifically, the treatment efficacy of surgical resection of pulmonary metastasis of meningioma remains unclear. To the best of our knowledge, 70 cases of pulmonary metastasis of meningioma have been reported to date in the English and Japanese literature [5, 6, 9, 10, 15, 16]. Among these, 30 cases have been reported with information about the outcomes of surgical resection for lung metastasis of cranial meningioma (Table 1) [5, 6, 15, 17–38]. A brief summary of clinical characteristics is as follows: the median age of the patients was 57 (range: 15–75) years, and women accounted for 46.7% (14 of 30 cases) of all patients. Moreover, WHO grade I, II, and III diseases accounted for 62.1% (18 of 29), 20.7% (6 of 29), and 17.2% (5 of 29), respectively, of all cases. All pulmonary lesions were resected during the first metastasectomy in 25 patients and the first and the second metastasectomies in 5 patients. The median survival time after the first pulmonary metastasectomy was 19 (range: 1–294) months. The 1-, 2-, and 5-year overall survival rates based on the integration of the reported 30 cases were 96.7%, 80.7%, and 40.4%, respectively (Fig. 4A). Further, WHO grade III meningioma was more likely to be associated with worse overall survival than WHO grade I meningioma (*P* = 0.082, Fig. 4B). Nevertheless, with consideration of publication and selection bias, small sample size, and heterogeneity of clinical and pathological background of the cases, which patients can benefit from pulmonary metastasectomy for lung metastasis of cranial meningioma remains unclear. Notably, a mortality case of metastatic meningioma, whose multiple pulmonary metastases and malignant pleural effusion seemed to be associated with respiratory failure, has been reported [39]. This indicates that prevention of multiple pulmonary metastasis would be beneficial for selected patients with metastatic meningioma. In the presented case, we decided to perform lung resection for the following reasons: (1) histological diagnosis to exclude the possibility of primary lung cancer; (2) possible benefit of disease control of metastatic meningioma by surgical removal of pulmonary metastasis, and (3) patient’s tolerability of lung resection. Further studies should address the question of whether surgical resection of pulmonary metastasis of meningioma can help improve patient prognosis.

**Table 1:** Outcomes of 30 patients undergoing surgical resection for lung metastasis of cranial meningioma

Author, year	Age^a^, sex	WHO grading of meningioma^b^	Interval^c^	No. of lung metastasis	Outcome^d^
Miller, 1985 [17]	61, M	I	N/A	1	22 months, alive
Le May, 1989 [18]	56, F	I	24 months	Multiple^e^	36 months, died
Kodama, 1991 [19]	61, F	I	228 months	4	44 months, alive
Kodama, 1992 [20]	30, M	I	144 months	1	3 months, alive
Adlakha, 1999 [15]	17, F	III	48 months	1	15 months, died
	70, F	I	N/A	2	48 months, died
	30, M	III	72 months	Multiple^e^	84 months, alive
Pramesh, 2003 [21]	29, F	I	96 months	2	24 months, alive
Knoop, 2004 [22]	53, M	I	N/A	1	24 months, alive
D’Aiuto, 2005 [23]	71, M	I	156 months	Multiple^e^	24 months, alive
Asioli, 2007 [24]	58, F	I	144 months	Multiple^e^	18 months, alive
Gladin, 2007 [25]	58, M	I	156 months	Multiple^e^	36 months, alive
	47, M	I	132 months	3	4 months, alive
Fulkerson, 2008 [26]	54, M	I	N/A	1	12 months, alive
Ishibashi, 2008 [27]	68, M	N/A	312 months	3	6 months, alive
Psaras, 2009 [28]	49, F	I	180 months	Multiple^e^	12 months, alive
Estanislau, 2009 [29]	75, M	II	24 months	1	30 months, died
Sazawa, 2009 [5]	66, F	I	N/A	1	14 months, died
Etienne-Mastroianni, 2010 [30]	58, M	III	N/A	4	20 months, died
Cheng, 2011 [31]	46, M	I	N/A	2	5 months, alive
Kanzaki, 2011 [32]	67, M	II	180 months	2	18 months, alive
Nakayama, 2014 [33]	25, F	I	N/A	4	294 months, alive
Sakamoto, 2014 [34]	15, F	II	60 months	3	6 months, alive
Tao, 2014 [35]	51, F	III	12 months	1	1 month, died
Chiarelli, 2015 [36]	68, M	I	N/A	1	36 months, alive
Frydrychowicz, 2015 [37]	72, F	II	60 months	1	72 months, alive
Sathirareuangchai, 2019 [16]	59, F	I	N/A	2	5 months, alive
Kimura, 2020 [6]	54, M	II	96 months	2	37 months, died
Mardani, 2021 [38]	37, F	III	0 month^f^	2	2 months, alive
Present case, 2021	75, M	II	24 months	1	8 months, alive

^a^Age at the time of the first pulmonary metastasectomy. ^b^The highest grade was presented if the original description included multiple grades. ^c^Interval between the detection of the primary tumor to the that of the first described pulmonary metastasis. ^d^Outcome of the initial pulmonary metastasectomy. ^e^The term “multiple” was used only if the original case report described the number of pulmonary metastases as “multiple” instead of specifying the exact number. ^f^The timing of detection of the primary meningioma and its pulmonary metastasis was simultaneous

## Conclusions

We report a rare and unique surgical case of solitary pulmonary metastasis of meningioma. Further investigation is necessary to establish the standardized treatment strategy for metastatic meningioma.

## Data Availability

The datasets supporting the conclusions of this article are included within the article.

## Abbreviation

WHO: World Health Organization
